# Choroidal Detachment after XEN Gel Stent Implantation

**DOI:** 10.1155/2021/6674505

**Published:** 2021-03-06

**Authors:** Carlo Alberto Cutolo, Letizia Negri, Sara Olivari, Francesca Cappelli, Carlo Enrico Traverso, Michele Iester

**Affiliations:** ^1^Clinica Oculistica, Department of Neuroscience Rehabilitation Ophthalmology Genetics Maternal and Child Health (DiNOGMI), University of Genoa, Genoa, Italy; ^2^IRCCS Ospedale Policlinico San Martino, Genoa, Italy

## Abstract

**Background:**

The purpose of this study is to estimate the incidence of choroidal detachment (CD) after XEN gel stent implant surgery and to evaluate the associated factors.

**Methods:**

We reviewed the clinical charts of 126 patients who underwent XEN implantation between March 1, 2016, and December 31, 2018 at the University Eye Clinic of Genoa. Ocular, demographics, and perioperative factor were registered and analysed. Univariate and multivariate logistic analysis were performed to investigate factors associated with CD occurrence.

**Results:**

Of the 126 patients, 25 (19.8%) developed a choroidal detachment after XEN gel stent implant surgery. The mean period between surgery and CD detection was 5.84 ± 1.77 days. The mean intraocular pressure (IOP) at the time of CD diagnosis was 6.4 ± 3.1 mmHg. Age (OR = 1.10, *p* < 0.019), early postoperative IOP (OR = 0.70, *p* < 0.001), and number of preoperative IOP-lowering drugs (OR = 5.70, *p* < 0.001) were significantly associated with CD presence. Complete resolution was observed in all the cases. Wide-field imaging and ultrasonography were useful tools to diagnose and follow up CD until resolution.

**Conclusions:**

When carefully investigated, CD is a relatively common complication after XEN gel stent implant procedure. Older age, lower postoperative IOP, and higher number of preoperative IOP-lowering drugs were significantly associated with the development of CD.

## 1. Introduction

Among the complication of glaucoma filtration surgeries, choroidal detachment (CD) is frequently observed. The estimated incidence varies from 3% to 34% and changes accordingly to the surgical technique to the ocular and the demographic characteristics of the patients and the diagnostic technique accuracy [1–6]. A limited CD can be asymptomatic, resolve spontaneously, and remain undetected if not adequately investigated. [7].

CD refers to an abnormal accumulation of either blood or serum in the suprachoroidal space, between the choroid and the sclera. In normal conditions, the suprachoroidal space is a potential volume because of the close apposition of the choroid to the sclera. In pathologic circumstances, due to alterations of ocular fluid dynamics, namely, the balance between hydrostatic and oncotic pressure, fluids can accumulate in this space. CD may be associated with hypotony of any aetiology or exudation of serum, most frequently caused by inflammation [8, 9]. Previous reports have identified many factors associated with CD after trabeculectomy and glaucoma drainage devices (i.e., age, hypotony, exfoliation, corneal thickness, pseudophakia, and hypertension) [3, 7, 10]. With the aim to reduce complications of glaucoma surgeries, novel devices that permitted more predictable filtration have been introduced in the clinical setting [11].

Among them, XEN gel stent is a 6 mm hydrophilic tube housed in a disposable preloaded handheld inserter explicitly designed for an ab-interno surgical implantation [12]. It decreases the intraocular pressure (IOP) by creating a permanent outflow pathway from the anterior chamber to the subconjunctival space through a scleral channel. CD has also been described after XEN gel stent implantation with incidences varying from 0 to 15% [13–18]. To the best of our knowledge, no studies have investigated the demographic and ocular characteristics associated with the occurrence of CD in patients implanted with the XEN gel stent. The purpose of this study was to estimate the occurrence of CD after XEN gel implantation surgery and to investigate the associated factors.

## 2. Materials and Methods

We retrospectively reviewed the medical records of 126 patients with glaucoma who underwent XEN gel stent implantation with mitomycin C (MMC) at Clinica Oculistica, San Martino Hospital IRCCS of Genoa, between March 2016 and December 2018. This study was part of a prospective, uncontrolled, consecutive case series [19]. The study followed the tenets of the Declaration of Helsinki, and written informed consent was obtained from all participants.

Eyes with the diagnosis of primary open-angle glaucoma or exfoliative glaucoma were included in the analysis. Other types of glaucoma or eyes with history of previous ocular surgeries other than uncomplicated phacoemulsification were excluded.

For all the patients, we considered demographic and ocular characteristics, namely, age, gender, systemic and ocular history, refractive status, intraoperative factors, complications during or after surgery, onset time of serous choroidal detachment, visual acuity (VA), and IOP were recorded and analysed. According to the clinical practice, postoperative visits were scheduled at days 1, 7, 14, and 30. Additional visits were scheduled when clinically required. Postoperatively, the following findings were recorded: VA, IOP, bleb status, wound leaks, anterior chamber (AC) depth, hyphema, anterior chamber inflammation, choroidal detachment, and other complications after surgery. In the case of numerical hypotony (IOP<6 mmHg), choroidal detachment detected during dilated fundus examination or in doubtful cases, ocular ultrasonography and ultra-wide-field imaging (Optos, Dunfermline, UK) were performed to confirm and follow up the disease. All the surgical procedures were performed by two surgeons (CET and MI). For all cases, 0.1 mL of MMC at the concentration of 0.2 mg/mL was injected subconjunctivally 30 minutes before the surgery. Surgical and postoperative managements were described in detail elsewhere [19, 20].

### 2.1. Statistical Analysis

In descriptive statistics, variables were summarized as means and standard deviation. Absolute value and percentage for frequency were used for categorical variables. Logistic regression analysis was performed to examine the association of the occurrence of postoperative CD and the ocular, demographic, and perioperative factors. A multivariate logistic regression model was built to investigate which characteristics better predict the development of CD. Variable selection for the multivariate model was based on statistical significance and biological plausibility. In case of bilateral surgery, only the first eye was considered for the analysis. A *p* value <0.05 was considered statistically significant. Statistical analysis was performed using Stata 15.1 (StataCorp LLC, College Station, TX).

## 3. Results

The demographic and ocular characteristics of the sample are summarized in Table 1.

Of the 126 patients included in the analysis, 25 (19.8%) developed a CD in the postoperative period. CD was diagnosed 5.84 ± 1.77 days after surgery and was serous in 22 (88%) eyes, while 3 (12%) eyes developed a haemorrhagic CD. The mean IOP at the time of diagnosis was 6.4 ± 3.1 mmHg. In all the cases, the CD did not extend to the posterior pole. VA returned to preoperative values in 22/25 (88%) CD cases within one month after surgery. In the remaining 3 (12%) cases, observed VA loss was less than 2 Snellen chart lines.

All the patients with CD received medical treatment with a topical steroid, cycloplegic eye drops, and oral glucocorticoids (prednisone 1 mg tapering off the dose until resolution). CD resolved successfully in 15.52 ± 7.15 days in all cases.

A shallow anterior chamber (AC) was noted in 9 (36%) of CD cases on day 1 and was managed with intracameral air and/or viscoelastic injection. Ultrasonography and ultra-wide-field imaging were used to document the diagnosis and follow-up the CD until resolution.

Table 2 reports the results of the univariate analysis. CD was significantly associated with older age (OR = 1.04; *p*=0.03), lower IOP in the first postoperative days (OR = 0.77; *p* < 0.001), and higher number of IOP-lowering drugs before surgery (OR = 3.49; *p* < 0.001).

Table 3 shows the results of the multivariate regression analysis. Age, IOP in the first postoperative days, and the number of IOP-lowering drugs remained significantly associated with CD occurrence.

## 4. Discussion

CD is a common complication of any filtering surgery, and hypotony and uveal inflammation are deemed to be notable causes [21, 22]. In our study, we have identified two characteristics of patients and one postoperative factor associated with CD development following the XEN gel stent implantation.

We observed that CD occurrence increased progressively with age, being more frequent in older patients. Jampel et al. also found that older patients were more likely to experience serous choroidal detachment after trabeculectomy in the collaborative initial glaucoma treatment study [23]. Older individuals might have relatively fragile connective tissue, which promotes fluid movement through the uveal tissue and the vascular barrier. The altered sclera of the older subject may also promote the compression of the vortex veins with an increase in the venous pressure and vessel leaking [3].

The present study identified lower postoperative IOP in the first week as a significative factor associated with CD occurrence. Low IOP is a well-known factor for CD after filtering surgeries [3, 10, 21, 23]. Xen gel stent has been designed to produce a pressure drop of about 8 mmHg [24]. Theoretical pressure drop can be calculated with the Hagen‐Poiseuille equation taking into account the physical dimension of the Xen and assuming a constant aqueous production. In the first postoperative period, aqueous production may be impaired by surgical trauma and antimetabolite toxicity to the ciliary body, reducing the theoretical pressure drop [22]. Moreover, a small amount of aqueous may flow around the implanted Xen. In a previous report, we found that low IOP in the postoperative factor is associated with better IOP control at 1 year [19]. The clinical success of glaucoma surgery is a trade-off between an IOP that is sufficiently low to slow the glaucomatous damages with no sign and symptoms of hypotony. In our study, we found that eyes treated with a higher number of preoperative IOP-lowering drugs were at higher risk for CD. There is a theoretical possibility that a higher number of preoperative medications together with postoperative hypotony increases ocular inflammation and exacerbate the end uveal effusion. Previous studies reported that IOP-lowering drugs were associated with CD occurrence in eyes with or without previous filtering intervention [25–30]. The pathophysiology of CD, induced or associated with medicaments, is not completely understood. It is deemed that low IOP and inflammation play a role in CD [26]. The ciliary body can be damaged by long-term use of drugs, and in case of surgery, aqueous production may be impaired, causing marked hypotony. On the other hand, prostaglandins (PGA) may permanently alter the level of collagen in the uveoscleral outflow pathway predisposing to postsurgical hypotony and may alter capillary permeability favouring uveal exudation [25, 31]. In addition, MMC may cause direct toxicity to the ciliary epithelium, resulting in hyposecretion [32, 33]. In our study, MMC concentration (0.2 mg/ml) used was identical in all the patients; hence, this variable was not explored in our analysis. In future research, it would be investigating the role of MMC as a risk factor of CD after Xen.

In our cases, all patients received medical treatment with topical, oral steroid, and cycloplegic and showed a complete resolution of the CD between 5 and 30 days after surgery. In few cases, when the AC was too shallow, an air or viscoelastic bubble was injected in the AC through the surgical paracentesis in the inferotemporal quadrant.

In our experience, CD following filter surgery using Xen gel stent was a transient complication and did not have a negative effect on the final outcome of the surgery.

This study is limited because of its retrospective nature. Randomized clinical trials on Xen gel stent are lacking. Further prospective studies could help to confirm the validity of our retrospective analysis.

In the case of CD diagnosis or suspect, we found clinically meaningful the use of wide-field imaging that permits noncontact and nondilated 200-degree imaging of the retina for diagnosis, documentation, and follow-up of CD. Unfortunately, no study has determined yet the accuracy of this technique to detect CD.

MIGS, as the Xen gel stent, has been the introduced to reduce postoperative complications and facilitate the surgical procedure [34]. Another advantage of MIGS is faster visual recovery and shorter surgical time compared to trabeculectomy. Postoperative complications may still limit the safety of these surgical procedures, and the identification of prognostic factors may permit to select the most suitable group of patients to offer the procedure.

## Figures and Tables

**Table 1:** Ocular, demographic, and perioperative characteristics.

Ocular and demographic characteristics	*N* = 126
Female	63 (50)
Age, years	71.3 ± 14.8
Glaucoma type	
POAG	95 (75.4)
XFG	31 (24.6)
Laterality of the eye	
Right	50 (39.7)
Left	76 (60.3)
History of cataract surgery	
No	67 (53.2)
Yes	59 (46.8)
Preoperative IOP	25.7 ± 8.2
Mean IOP, 1 to 7 days after surgery	9.8 ± 5.2
Type of procedure	
Standalone	96 (76.2)
Combined	30 (23.8)
Preoperative IOP-lowering drugs, n	3 ± 0.9

Data are mean ± sd or number (%). POAG = primary open-angle glaucoma; XFG = exfoliative glaucoma; IOP= intraocular pressure.

**Table 2:** Univariate analysis results (factors associated with the occurrence of choroidal detachment after Xen gel stent surgery).

Predictor variable	Odds ratio	95% CI	*P* Value
Age, years	1.04	1.00 to 1.10	**0.03**
Preoperative IOP, mmHg	1.03	0.97 to 1.08	0.35
Preoperative IOP-lowering drugs, *n*	3.49	1.94 to 6.28	**<0.001**
Glaucoma type	1.17	0.79 to 1.73	0.43
POAG	1.00		
XFG	3.27	0.92 to 11.58	
Mean IOP, 1 to 7 days after surgery	0.77	0.66 to 0.89	**<0.001**
Procedure	0.55	0.17 to 1.75	0.31
Standalone	1.00		
Combined	0.59	0.18 to 1.90	

IOP = intraocular pressure; POAG = primary open-angle glaucoma; XFG = exfoliative glaucoma. Bold means that the value is statistically significant.

**Table 3:** Multivariate analysis results (factors associated with the occurrence of choroidal detachment after Xen gel stent surgery).

Predictor variable	Odds ratio	95% CI	*P* value
Age, years	1.10	1.01 to 1.19	**0.019**
Preoperative IOP-lowering drugs, n	0.99	0.89 to 1.09	0.855
Preoperative drugs, *n*	5.70	1.98 to 16.36	**0.001**
Glaucoma type	1.23	0.59 to 2.58	0.576
Mean IOP, 1 to 7 days after surgery	0.70	0.56 to 0.87	**0.001**
Procedure	0.72	0.10 to 5.27	0.749

IOP = intraocular pressure. Bold means that the value is statistically significant.

## Data Availability

The data used to support the findings of this study are included within the article.

## References

[1] Mills K. B. (1981). Trabeculectomy: a retrospective long-term follow-up of 444 cases. *British Journal of Ophthalmology*.

[2] Gedde S. J., Schiffman J. C., Feuer W. J., Herndon L. W., Brandt J. D., Budenz D. L. (2012). Treatment outcomes in the tube versus trabeculectomy (TVT) study after five years of follow-up. *American Journal of Ophthalmology*.

[3] Haga A., Inatani M., Shobayashi K., Kojima S., Inoue T., Tanihara H. (2013). Risk factors for choroidal detachment after trabeculectomy with mitomycin C. *Clinical Ophthalmology*.

[4] Brubaker R. F., Pederson J. E. (1983). Ciliochoroidal detachment. *Survey of Ophthalmology*.

[5] Shirato S., Kitazawa Y., Mishima S. (1982). A critical analysis of the trabeculectomy results by a prospective follow-up design. *Japanese Journal of Ophthalmology*.

[6] Buffault J., Graber M., Bensmail D. (2020). Efficacy and safety at 6 months of the XEN implant for the management of open angle glaucoma. *Scientific Reports*.

[7] Shin D. Y., Jung K. I., Park H. Y. L., Park C. K. (2020). Risk factors for choroidal detachment after ahmed valve implantation in glaucoma patients. *American Journal of Ophthalmology*.

[8] Bellows A. R., Chylack L. T., Hutchinson B. T. (1981). Choroidal detachment. *Ophthalmology*.

[9] Brav S. S. (1961). Serous choroidal detachment. *Survey of Ophthalmology*.

[10] Iwasaki K., Kakimoto H., Arimura S., Takamura Y., Inatani M. (2020). Prospective cohort study of risk factors for choroidal detachment after trabeculectomy. *International Ophthalmology*.

[11] Lavia C., Dallorto L., Maule M., Ceccarelli M., Fea A. M. (2017). Minimally-invasive glaucoma surgeries (MIGS) for open angle glaucoma: a systematic review and meta-analysis. *PLoS One*.

[12] Wagner F. M., Schuster A. K.-G., Emmerich J., Chronopoulos P., Hoffmann E. M. (2020). Efficacy and safety of XEN®-Implantation vs. trabeculectomy: data of a “real-world” setting. *PLoS One*.

[13] Karimi A., Lindfield D., Turnbull A. (2019). A multi-centre interventional case series of 259 ab-interno Xen gel implants for glaucoma, with and without combined cataract surgery. *Eye*.

[14] Smith M., Charles R., Abdel-Hay A. (2019). 1-year outcomes of the Xen45 glaucoma implant. *Eye*.

[15] Grover D. S., Flynn W. J., Bashford K. P. (2017). Performance and safety of a new ab interno gelatin stent in refractory glaucoma at 12 months. *American Journal of Ophthalmology*.

[16] Gillmann K., Bravetti G. E., Rao H. L., Mermoud A., Mansouri K. (2020). Combined and stand-alone XEN 45 gel stent implantation: 3-year outcomes and success predictors. *Acta Ophthalmologica*.

[17] Reitsamer H., Sng C., Sng C. (2019). Two-year results of a multicenter study of the ab interno gelatin implant in medically uncontrolled primary open-angle glaucoma. *Graefe’s Archive for Clinical and Experimental Ophthalmology*.

[18] De Gregorio A., Pedrotti E., Stevan G., Bertoncello A., Morselli S. (2018). XEN glaucoma treatment system in the management of refractory glaucomas: a short review on trial data and potential role in clinical practice. *Clinical Ophthalmology*.

[19] Cutolo C. A., Iester M., Bagnis A. (2020). Early postoperative intraocular pressure is associated with better pressure control after XEN implantation. *Journal of Glaucoma*.

[20] Olivari S., Cutolo C. A., Negri L. (2019). XEN implant fracture during needling procedure. *Journal of Glaucoma*.

[21] Berke S. J., Bellows A. R., Shingleton B. J., Richter C. U., Hutchinson B. T. (1987). Chronic and recurrent choroidal detachment after glaucoma filtering surgery. *Ophthalmology*.

[22] Toris C. B., Pederson J. E. (1987). Aqueous humor dynamics in experimental iridocyclitis. *Investigative Ophthalmology & Visual Science*.

[23] Jampel H. D., Musch D. C., Gillespie B. W., Lichter P. R., Wright M. M., Guire K. E. (2005). Perioperative complications of trabeculectomy in the collaborative initial glaucoma treatment study (CIGTS). *American Journal of Ophthalmology*.

[24] Sheybani A., Reitsamer H., Ahmed I. I. K. (2015). Fluid dynamics of a novel micro-fistula implant for the surgical treatment of glaucoma. *Investigative Opthalmology & Visual Science*.

[25] Pedersen O. (1980). Increased vascular permeability in the rabbit iris induced by prostaglandin E1. *Albrecht von Graefes Archiv für Klinische und Experimentelle Ophthalmologie*.

[26] Vela M. A., Campbell D. G. (1985). Hypotony and ciliochoroidal detachment following pharmacologic aqueous suppressant therapy in previously filtered patients. *Ophthalmology*.

[27] Alimgil M. L., Benian Ö. (2001). Choroidal effusion and shallowing of the anterior chamber after adjunctive therapy with latanoprost in a trabeculectomized patient with angle closure glaucoma. *International Ophthalmology*.

[28] Marques Pereira M. L., Katz L. J. (2001). Choroidal detachment after the use of topical latanoprost. *American Journal of Ophthalmology*.

[29] Callahan C., Ayyala R. S. (2003). Hypotony and choroidal effusion induced by topical timolol and dorzolamide in patients with previous glaucoma drainage device implantation. *Ophthalmic Surgery, Lasers & Imaging Official Journal of the International Society for Imaging*.

[30] Goldberg S., Gallily R., Bishara S., Blumenthal E. Z. (2004). Dorzolamide-induced choroidal detachment in a surgically untreated eye. *American Journal of Ophthalmology*.

[31] Sagara T., Gaton D. D., Lindsey J. D., Gabelt B. T., Kaufman P. L., Weinreb R. N. (1960). Topical prostaglandin F2alpha treatment reduces collagen types I, III, and IV in the monkey uveoscleral outflow pathway. *Archives of Ophthalmology Chic*.

[32] Mietz H. (1996). The toxicology of mitomycin C on the ciliary body. *Current Opinion in Ophthalmology*.

[33] Levy J., Tessler Z., Rosenthal G. (2001). Toxic effects of subconjunctival 5-fluorouracil and mitomycin C on ciliary body of rats. *International Ophthalmology*.

[34] Mansouri K., Guidotti J., Rao H. L. (2017). Prospective evaluation of standalone XEN gel implant and combined phacoemulsification-XEN gel implant surgery: 1-year results. *Journal of Glaucoma*.

